# Flying microbes—survival in the extreme conditions of the stratosphere during a stratospheric balloon flight experiment

**DOI:** 10.1128/spectrum.03982-23

**Published:** 2024-06-13

**Authors:** Tim Heitkämper, Raphael Roth, Stephan Harteneck, Felix Berger, Sonya Salam, Chunyu Fey-Du, Christopher Flöck, Niclas Tschierske, Vincent Vonderbank, Alexander Martin, Sebastian Erren, Joel Zimmermann, Mike Lutz, Katharina Kujala

**Affiliations:** 1 FH Aachen, Faculty 03 Chemistry and Biotechnology/Faculty 10 Energy Technology, Jülich, Germany; 2 FH Vorarlberg, Faculty of Business Administration, Dornbirn, Austria; 3 University of Oulu, Water, Energy and Environmental Engineering Research Unit, Oulu, Finland; Dominican University New York, Orangeburg, New York, USA

**Keywords:** stratosphere, UV radiation, survival, *Bacillus subtilis*, microbial communities

## Abstract

Earth’s stratosphere is characterized by hypobaric conditions, low temperatures, and high intensities of ultraviolet (UV) and cosmic radiation as well as low water and nutrient availability. While it is not considered a permanent habitat for microorganisms, they can be transported to the stratosphere by storms, volcanic action, or human activity. The impact of those extreme conditions on microorganisms and their survival were tested by sending a sample gondola to the stratosphere. The sample gondola was built to allow exposure of *Bacillus subtilis* endospores at different angles to the sun. It moreover had holders for three environmental samples to test the effect of stratospheric conditions on complex microbial communities. The gondola attached to a stratospheric balloon was launched near Kiruna, Sweden, ascended to ~25 km, and drifted eastward for ~200 km. Samples were exposed to pressures as low as 2 kPa and temperatures as low as −50°C as well as high UV radiation. Survival rates of *B. subtilis* were determined by comparing the numbers of colony-forming units (CFUs) for the different exposure angles. Survival was negatively correlated with exposure angle, indicating the significant impact of UV radiation. The effect of stratospheric conditions on environmental samples was assessed by comparing most probable numbers, microbial community composition, and substrate-use profiles to controls that had stayed on the ground. Cultivation was possible from all samples with survival rates of at least 1%, and differences in community composition were observed. Survival of environmental microorganisms might have been supported by the sample matrix, which provided protection from radiation and desiccation.

**IMPORTANCE:**

Earth's stratosphere is a hostile environment that has challenged microbial survival. We set out to test the effect of stratosphere exposure on survival of single species (*Bacillus subtilis*) and complex microbial communities from soils and sediment. *B. subtilis* survival was strongly impacted by sun exposure, i.e., ultraviolet (UV) radiation, with only 1% survival at full sun exposure. Complex microbial communities had high survival rates, and the soil or sediment matrix may have provided protection against radiation and desiccation, supporting the survival of environmental microorganisms.

## INTRODUCTION

Earth’s stratosphere, extending from ~10 km to 50 km above sea level, is an extremely hostile environment, and the extent to which life is possible in the stratosphere is likely low. Stressors in the stratosphere include ultraviolet (UV) and cosmic radiation, cold temperatures ranging from −50°C in the lower to −15°C in the upper stratosphere, hypobaric pressure of < 100 mbar, desiccation, starvation, and ozone (1). Natural phenomena such as volcanic eruptions or dust storms as well as human activity such as air travel, satellites, or meteorological balloons can transport microorganisms into the stratosphere (1). If and how long organisms survive stratospheric conditions are still only incompletely resolved, and the contribution of different stressors is likewise uncertain. While dispersal of microorganisms in the troposphere, i.e., the lower layer of the atmosphere, over longer distances is common, e.g., attached to sand particles that are uplifted during desert dust storms (2), dispersal of microorganisms through the stratosphere is more uncertain. Aerosol sampling in the stratosphere has revealed the presence of microbial DNA as well as viable microorganisms (1, 3, 4). While viable cells have been recovered from up to a height of 41 km (3, 5), the numbers retrieved from higher altitudes are significantly lower than those retrieved in the convective boundary layer (3).

The extent to which microorganisms can persist in the stratosphere is highly variable and depends on the microbial species: some species are completely inactivated by stratospheric conditions with or without UV exposure, while others can withstand stratospheric conditions fairly well (6–8). However, reduction in the number of viable cells and delayed growth after stratosphere exposure are also frequently observed for more resistant species (6–8). There are many ways in which microbes can resist stratospheric conditions. Endospore formers such as *Bacillus subtilis* can survive in the stratosphere in a dormant state protected by their endospore capsule. Moreover, increased exposure to sunlight selects for pigment producers, as pigments help shield the cell from harmful UV radiation (9, 10). Aggregate formation, as observed in *Deinococcus* spp., can also aid survival by shielding cells inside the aggregate from direct radiation and desiccation (1, 4). *Deinococcus* spp. are moreover famous for their extremely robust DNA repair mechanisms, which allow for the quick repair of radiation-damaged genetic material (11). While microorganisms may be able to survive in the stratosphere even for extended periods, they may not necessarily be metabolically active. RNA is often used as an indicator of active microorganisms, and RNA has been isolated from samples collected in the lower atmosphere (e.g., on mountain tops or in clouds), indicating at least a basic level of microbial activity (12, 13). However, whether microorganisms can be active over long periods in the higher stratosphere needs still further investigation.

In our student-initiated project, Experiment on Outliving Microorganisms under Stratospheric Environment (EOSTRE), we set out to explore the impact of stratospheric conditions on microbial survival and functioning by sending endospores of *Bacillus subtilis* as well as complex environmental microbial communities to the stratosphere using a zero-pressure balloon. Our project involved students from diverse fields of science and technology studying at the FH Aachen–University of Applied Sciences, who conceived the study, acquired funding, and planned and conducted most of the experiments, including design and construction of the sample gondola, flight data collection, and cultivation experiments. The results of this study demonstrate the great value of the team effort of a student team with diverse backgrounds and can be used as a baseline for potential future efforts of our team.

## RESULTS AND DISCUSSION

### Stratospheric flight

**Fig 1:**
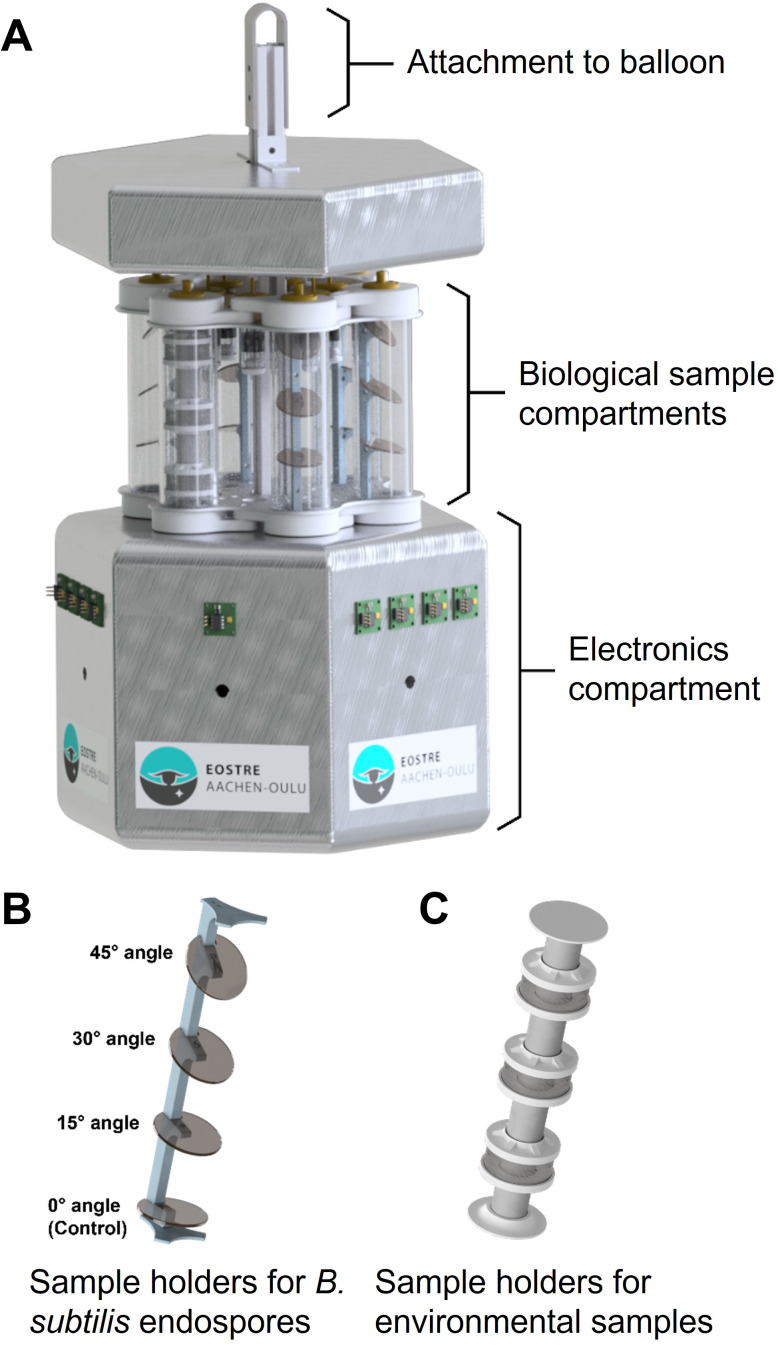
Sample gondola (**A**) with sample holders and a separate compartment for sensors and other electronics. Sample holders were placed in UV-transparent tubes. Five sample holders for *B. subtilis* endospores (**B**) and one sample holder for environmental samples (**C**) were mounted onto the gondola.

**Fig 2:**
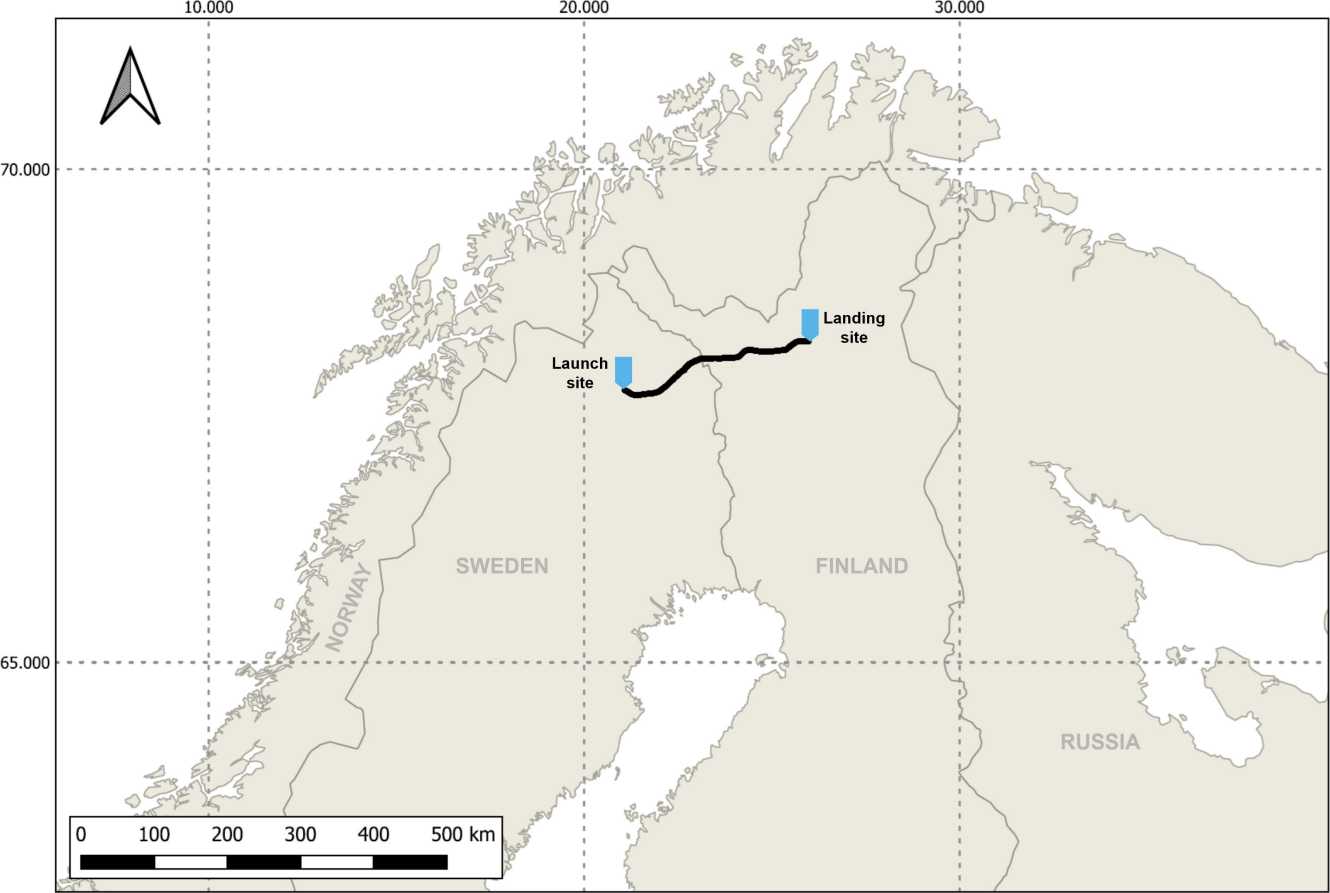
Flight path of the stratospheric balloon. The balloon was launched from the Swedish Space Corporation facility in Kiruna, Sweden, on 3 November 2020 and landed near Inari, Finland, after it had traversed a distance of approximately 200 km at up to 25 km height. The map was drawn using QGIS, an open-source program. The “world map” is one of the basemaps in the program.

**Fig 3:**
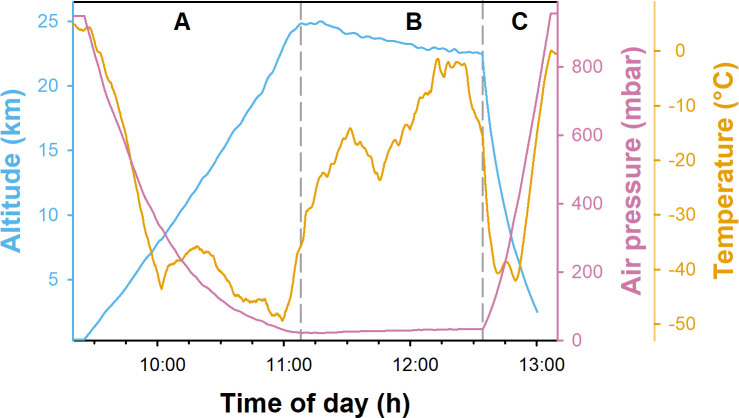
Altitude, air pressure, and external temperature recorded during the stratospheric balloon flight. The balloon flight was executed on 3 November 2020. The different phases of the flight are indicated: A = ascent, B = floating, and C = descent.

**Fig 4:**
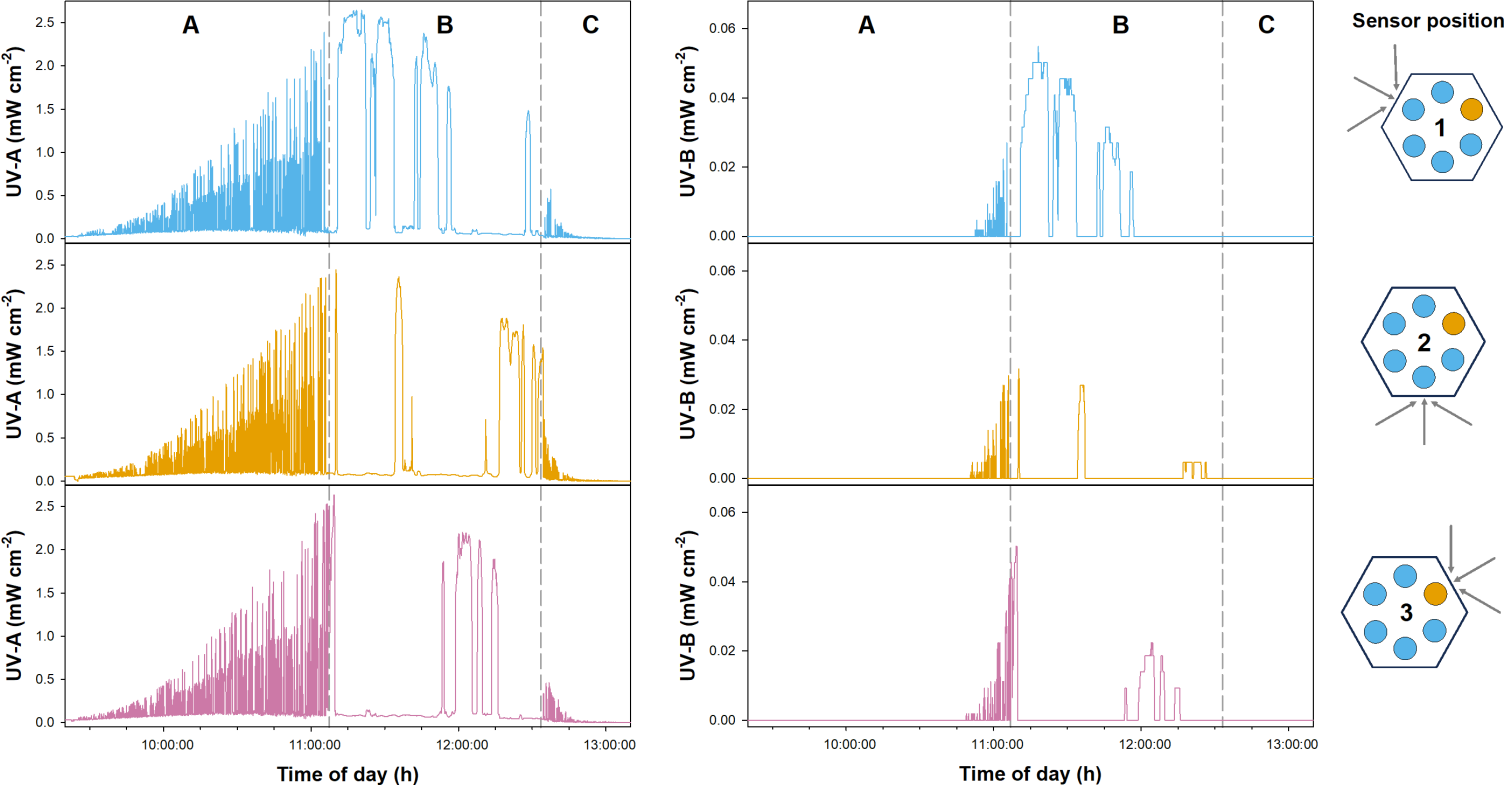
Incoming UV radiation for different sides of the gondola. UV-A and UV-B radiation measurements from sensors attached to the outside of the electronics compartment (Fig. 2) are shown. The gondola was also equipped with sensors for UV-C radiation (capturing UV radiation from a 110°sector), which was not detected. The different phases of the flight are indicated: A = ascent, B = floating, and C = descent. The schematic drawing on the right indicates the positioning of the sample holders relative to the sensor: blue = holder for *B. subtilis* endospores; yellow = holder for soil samples. Cumulative radiation doses were calculated for each sensor. Cumulative UV-A radiation amounted to 6.25·10^3^, 3.86·10^3,^ and 3.91·10^3^ mJ·cm^−2^ for sensors 1, 2, and 3, respectively. Cumulative UV-B radiation amounted to 68.8, 9.5, and 20.6 mJ·cm^−2^ for sensors 1, 2, and 3, respectively.

A sample gondola carrying six sample holders with biological material (Fig. 1) was launched on a stratospheric flight using a zero-pressure balloon. The total flight duration was approximately 3.5 h, during which the gondola traveled a total distance of 200 km (Fig. 2). At the final float altitude of approximately 25 km, the atmospheric pressure was 20 to 40 mbar (Fig. 3). Temperatures varied from −50 to −5°C, and higher temperatures toward the end of the float period were likely caused by sun warming. During the ascent and descent, the gondola experienced pronounced rotation, as indicated by the measured angular orientation (Fig. S1). In consequence, the amount of received UV radiation was also strongly fluctuating at different sides of the gondola (Fig. 4). Once the gondola reached flight height, the orientation of the gondola became more stable (Fig. S1), and the measured UV-A and UV-B radiation doses were likewise more stable. Due to this, the cumulative radiation dose differed between the sensors: sensor 1 received the highest overall dose with 6.25·10^3^ and 68.8 mJ·cm^−2^ of UV-A and UV-B radiation, respectively, while UV-A and UV-B radiation doses measured for sensors 2 and 3 were approximately 40% and 70%–80% lower, respectively (Fig. 4). In consequence, the different sample holders were likewise exposed to different overall doses.

### Impact of stratospheric conditions and sun exposure on microbial survival

**Fig 5:**
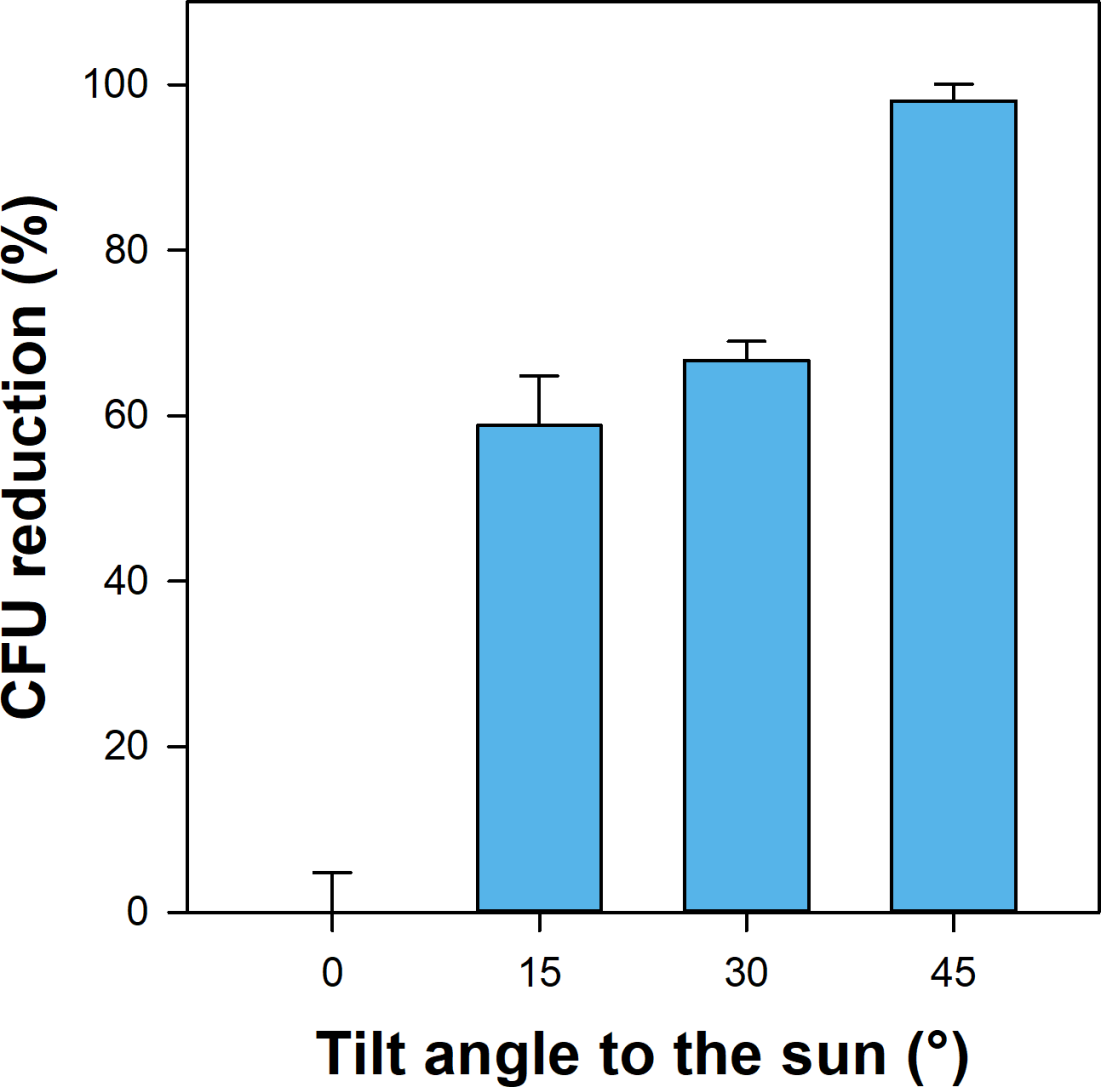
Impact of sun exposure on survival rates of *B. subtilis* endospores. CFU counts of endospores that were on the stratospheric flight with different exposure angles to the sun (15° = lowest exposure; 45° = highest exposure) were compared to CFU counts of endospores that had no direct sun exposure (0°= no exposure). Means and standard errors of four replicates are displayed.

Survival of bacteria in the stratosphere is challenged by a combination of different stressors. Our study assessed microbial survival after 3.5 h of stratosphere exposure for spores of *B. subtilis* as well as complex microbial communities in soils. UV radiation has been shown to have the greatest impact on the survival of *B. subtilis* spores in stratosphere simulation experiments (14). In our study, the impact of UV radiation on spore survival was assessed by exposing *B. subtilis* endospores at different exposure angles, ranging from no direct sun exposure (0° tilt angle) to maximum exposure (45° tilt angle). When compared to endospores with no direct sun exposure, increasing exposure angles strongly impacted *B. subtilis* survival, as the number of colony-forming units (CFUs) decreased with increasing exposure angle to ~1% of the CFUs at a 0° tilt angle (Fig. 5). This is in agreement with the findings of a simulation experiment that found that >99% of *B. subtilis* spores were killed after 6 hours of UV exposure (14). Other stressors such as low atmospheric pressure, low temperature, and desiccation, which were similar at all exposure angles, might also have played a role in reducing spore viability. *B. subtilis* endospores are known for their ability to resist stressors such as desiccation, heat, or toxic chemicals (15). There is, moreover, evidence that hypobaric pressure has only a minor impact on growth and survival of *B. subtilis* (16). Indeed, simulation experiments have found no impact of stratospheric conditions on spore survival when spores were not exposed to UV radiation (14, 17).

**Fig 6:**
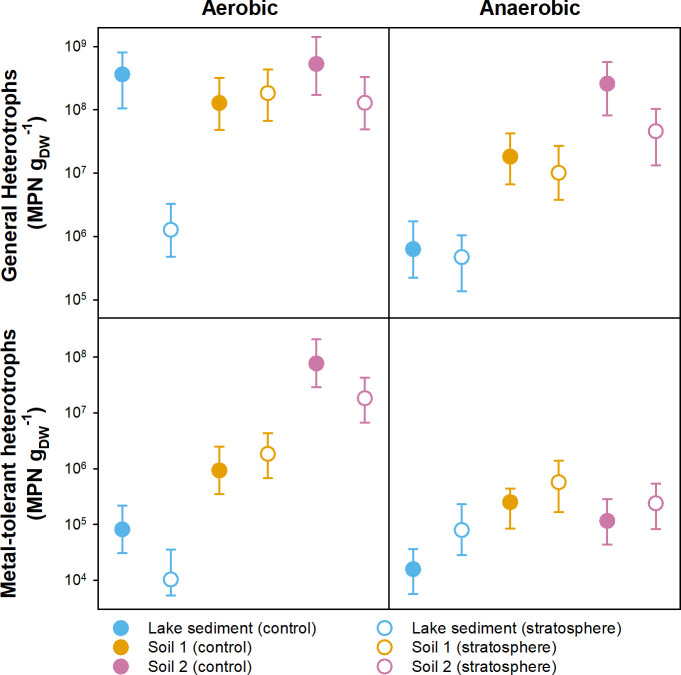
Impact of stratospheric flight on most probable number (MPN) counts of general and metal-tolerant aerobic and anerobic heterotrophs. MPNs based on six replicates and 95% confidence intervals are displayed.

As the extent of the negative impact on microbial survival varies between species, the impact of stratospheric conditions on survival in complex microbial communities will be largely impacted by the species composition within that community. Soil microbial communities in general have high taxonomic and functional diversity, which makes them rather resilient to disturbances (18). Thus, in our study, we tested the impact of stratospheric conditions on three environmental samples, two peat soil samples and one lake sediment sample. We compared most probable number (MPN) counts of stratosphere-exposed samples and earthbound controls for these three samples and found inconsistent effects of the stratospheric flight on survival rates (Fig. 6). For general aerobic heterotrophs, MPNs ranged from 10^8^ to 10^9^ cells g_DW_^−1^ in all earthbound controls (Fig. 6). No effect of stratospheric conditions was seen for the two peat soils (>10^8^ cells g_DW_^−1^ after stratospheric flight), while for the lake sediment, there was a pronounced reduction in culturable cell number (10^6^ cells g_DW_^−1^). Most probable numbers of general anerobic heterotrophs differed between different soils/sediment in the controls (10^6^ to 10^9^ cells g_DW_^−1^), with the lowest number of culturable anerobic heterotrophs in the lake sediment, but MPNs were largely unaffected by the stratospheric flight.

Most probable numbers of both aerobic and anerobic metal-tolerant heterotrophs were lower than those of general heterotrophs in all soils/sediment, with slightly higher MPN counts in peat soils than in the lake sediment (Fig. 6). Metal-tolerant microorganisms often show high UV tolerance (19, 20), and metal-tolerant microorganisms might thus be better equipped in general for survival in the stratosphere. Moreover, metal tolerance is often co-occurring with antibiotic resistance, and UV- and metal-tolerant microorganisms could thus be involved in the dispersal of antibiotic resistance (21). On the other hand, stratospheric UV exposure has been reported to negatively affect antibiotics and metal resistance in several bacterial strains (7). For our samples, no consistent trend for the effect of stratospheric conditions on growth of metal-tolerant heterotrophs was observed (Fig. 6), highlighting that complex microbial communities might host metal-tolerant microorganisms with and without UV tolerance.

### Impact of stratospheric conditions on the composition and functioning of complex microbial communities

**Fig 7:**
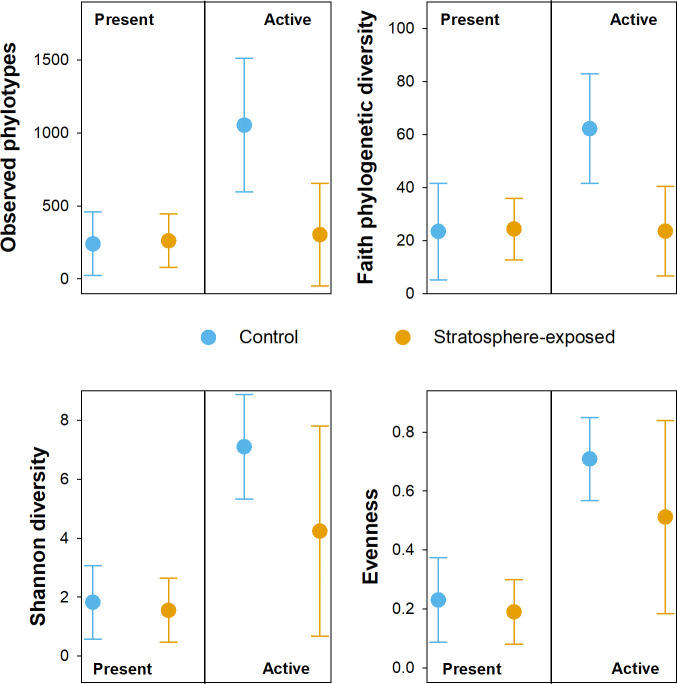
Impact of stratospheric flight on the diversity of the present (DNA-based 16S amplicon sequencing) and active (RNA-based 16S amplicon sequencing) microbial communities in samples exposed to the stratosphere (stratosphere-exposed) and samples kept in controlled conditions on the ground (control). Averages and standard deviation of three samples are shown.

**Fig 8:**
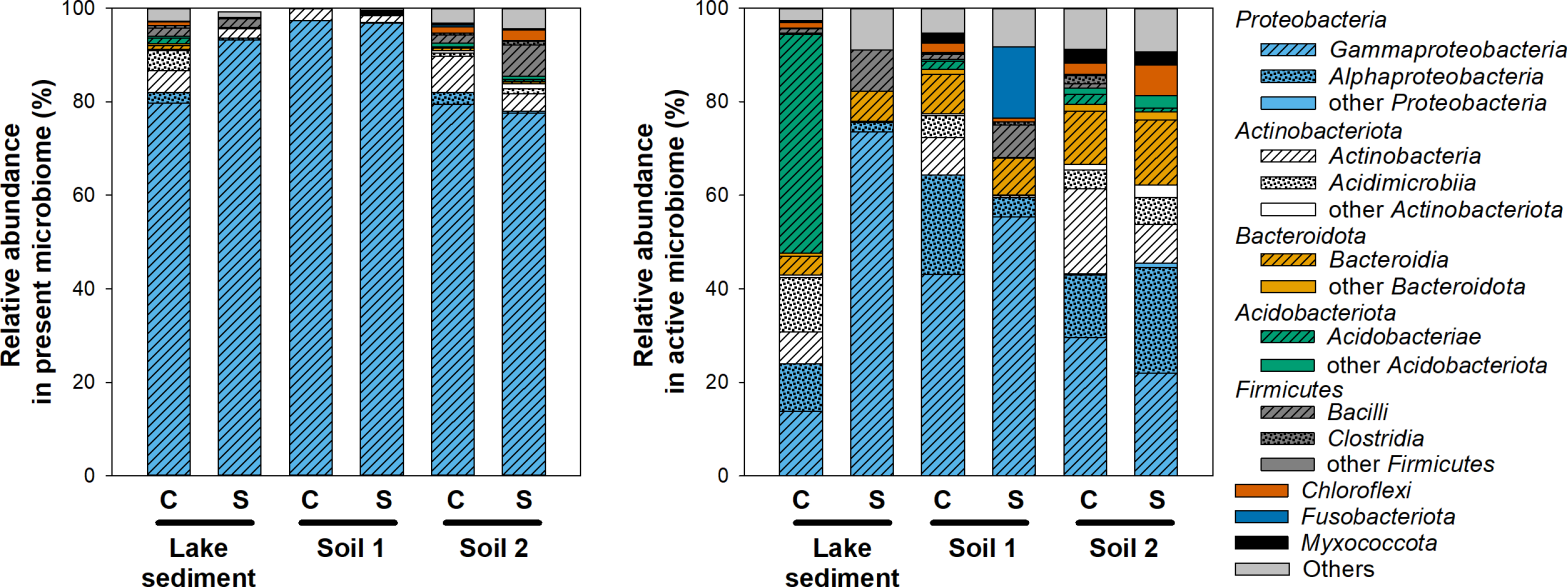
Class-level composition of the present (DNA-based 16S amplicon sequencing; left) and active (RNA-based 16S amplicon sequencing; right) microbial communities in samples exposed to the stratosphere (S) and samples kept in controlled conditions on the ground (C).

The impact of stratosphere exposure on microbial community composition was assessed through 16S rRNA and 16S rRNA gene amplicon sequencing of the stratosphere-exposed and earthbound control samples, which yielded 71,000 ± 29,000 quality-filtered sequences per sample. Overall, earthbound and stratosphere-exposed samples had similar numbers of detected amplicon sequencing variants (ASVs), similar Faith phylogenetic and Shannon diversity, and similar evenness on DNA-level, while on RNA-level, these diversity indicators were slightly lower in stratosphere-exposed than in earthbound samples (Fig. 7). This indicates that stratospheric conditions impacted active microbial communities, as only more resistant microbes would have been able to be active under these conditions. DNA-level analysis, which was used as an indicator for all microbes present in the samples, cannot discriminate between viable and dead microbial cells, and it is feasible that there might be more dead cells post-flight, which would make the impact of stratospheric conditions less discernible. Soil minerals protect organic compounds such as nucleotides from radiation (22), and this shielding from direct UV exposure might have helped DNA to persist even after cell death. A recent study has indeed demonstrated that only 10%–30% of DNA-containing cells sampled in the stratosphere were viable, and the percentage decreased with increasing height (3). On DNA level, microbial communities were strongly dominated by *Proteobacteria* (mostly *Gammaproteobacteria*) in all but one sample (≥78% relative abundance), followed by *Actinobacteriota* (2% to 18% relative abundance) and *Bacteroidota* (0% to 16% relative abundance) (Fig. 8). *Proteobacteria* and *Actinobacteria* have previously been isolated from samples collected in the stratosphere (3), highlighting the potential of these phyla to withstand the extreme conditions in the stratosphere. For all soils, differences in community composition in stratosphere-exposed and earthbound control samples were small (Fig. 8; Fig. S2). On RNA-level, which was used as an indicator for active microbes present in the samples, microbial communities were dominated by *Alphaproteobacteria* and *Gammaproteobacteria* (2% to 22% and 13% to 82% relative abundance, respectively), *Actinobacteria* (up to 18% relative abundance), *Acidimicrobiia* (up to 12% relative abundance), and *Bacteroidia* (up to 14% relative abundance) (Fig. 8). In the control lake sample, *Acidobacteriae*, which were of low abundance in all other samples, had a relative abundance of 47% (Fig. 8). In the stratosphere-exposed lake sample, however, the relative abundance of *Acidobacteriae* was low (<1%), indicating that the activity of this group was strongly impacted by stratospheric conditions.

**Fig 9:**
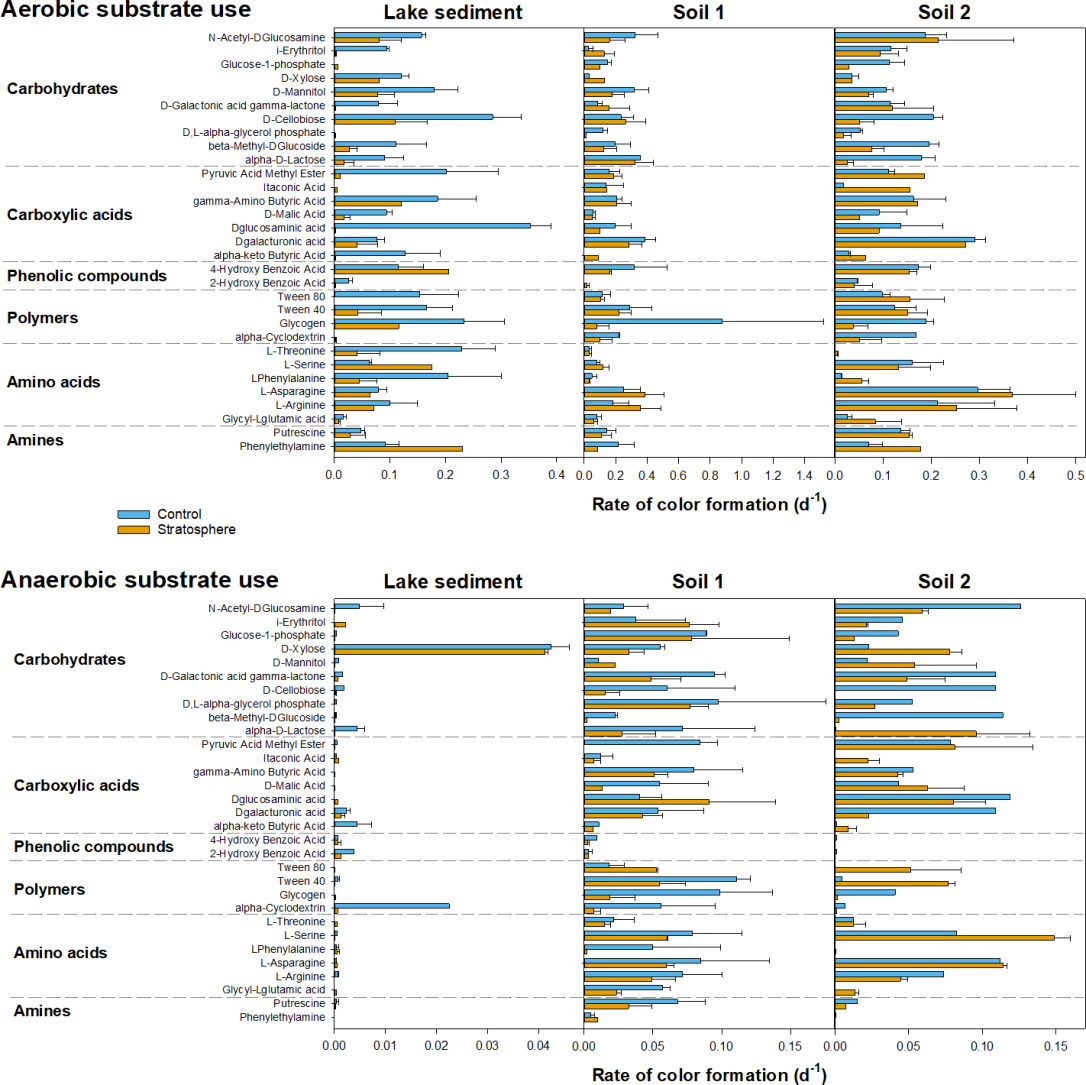
Impact of stratospheric flight on aerobic and anerobic substrate use in Ecoplates. Rate of color formation was determined based on repeated optical density (OD) measurements at 590 nm.

Aerobic heterotrophs were able to utilize a variety of carbon substrates in control and stratosphere-exposed samples (Fig. 9). While many substrates were used aerobically by all samples, the lake sediment showed much lower anerobic substrate use rates than the two peat soils for both control and stratosphere-exposed samples. D-Xylose was the only substrate that was used well by both, while N-acetyl-D-glucosamine, alpha-D-lactose, and alpha-cyclodextrin were only used well in the control (Fig. 9). This is in agreement with the low numbers of culturable anerobic heterotrophs in the lake sediment (Fig. 6). The effect of stratosphere exposure on substrate use was variable. In the lake sediment, stratosphere exposure reduced both aerobic and anerobic use rates of many substrates by up to 150 x (Fig. 9; Fig. S3). This is in agreement with studies that have reported negative impacts of UV exposure on substrate use and fermentation potential in Gram-positive and -negative strains (7). In soil 1, aerobic and anerobic use of many substrates was likewise reduced in the stratosphere-exposed sample, while in soil 2, improved substrate use was detected in the stratosphere-exposed sample, especially for carboxylic acids and amino acids (Fig. 9; Fig. S3). UV radiation can lead to photodegradation of organic matter and alter the composition of the remaining material (23, 24), and recalcitrant compounds can be broken down into more accessible compounds, which in turn stimulate microbial decomposition (24). It is thus possible that different groups of microorganisms were activated during the stratospheric flight, which might explain the observed differences in MPNs, microbial community composition, and substrate use.

### Conclusions and outlook

In this student-initiated project, we were able to design and assemble a gondola to carry multiple types of biological samples and send this gondola onto a ~3.5-h stratospheric flight. The results we obtained indicate that (i) microorganisms (pure cultures as well as complex microbial communities) can survive in the stratosphere, and recultivation after landing is successful; (ii) the impact of stratospheric conditions on complex microbial communities was inconclusive, ranging from no pronounced effect in some cases (e.g., anerobic heterotroph MPN counts, and DNA-based microbial community composition) to strong negative impacts in others (e.g., aerobic heterotroph MPN counts in lake sediment); and (iii) metabolic potential can be strongly affected by stratosphere exposure.

We hope to follow up the EOSTRE flight with further experiments that would include more in-depth studies of impacts on microbial community composition and activity (including different sample types and replicated testing); activity estimations during the flight, as opposed to after landing; and testing different shielding mechanisms that might protect earth-borne microbial communities during stratospheric travel.

## MATERIALS AND METHODS

### Stratospheric flight

The stratospheric flight was realized using a zero-pressure balloon to which a sample gondola was attached. The balloon was launched on 3 November 2020 from the facilities of the Swedish Space Corporation (SSC) in Kiruna, Sweden (67.89°N; 21.08°E), at 09:25 local time. It ascended over the course of 1.5 h to a final float height of 25 km, where it floated eastward for approximately 1.5 h, after which the gondola was separated from the balloon by cutting the cable holding the gondola with a pyrocutter triggered by a radio signal sent from the operator at the SSC, and the gondola descended to Inari, Finland (68.35°N; 26.08°E), with a parachute (Fig. 1). The gondola was recovered from the landing site within 24 h of landing.

### Gondola and sensors

A lightweight sample gondola (3 kg) was constructed to hold the biological samples, a variety of sensors, microcontrollers, and batteries (Fig. 1A). The mounted sensors monitored temperature; air pressure; angular orientation of the gondola; and UV-A, UV-B, and UV-C radiation. UV sensors were purchased from commercial suppliers (UV-A = ML811, Lapis Semiconductor Co., Ltd., Japan; UV-B/UV-C = GUVB-11SD/GUVC-T21GH, GenUV, South Korea) and detected radiation at wavelengths ranging from 280 to 390 nm (max. at 365 nm), 240–320 nm (max. at 300 nm), and 220–280 nm (max. at 254 nm). Three sensor packages for UV radiation were located equally spaced around the gondola. The number of sensors was chosen because each sensor had a cone-shaped field of vision with an opening angle of 55° to the normal axis of the sensor, so one sensor can observe a 110° field of the sky. Placed on every second face of the gondola, the sensors were thus catching radiation of almost the entire circle around the gondola, with 330° of observation. Biological samples were placed in six hexagonally arranged UV-transparent tubes made of polymethyl methacrylate (PMMA). Sterile filters (0.2 µm) at the top of the tubes ensured ventilation and pressure compensation of the otherwise sealed tubes. These sterile filters were encased by UV-resistant plastic and were thus shielded from direct UV exposure.

### Preparation and mounting of biological materials

Two different types of biological material were used in the stratospheric flight experiment: *Bacillus subtilis* endospores to demonstrate the survival rates of a resistant pure culture as well as environmental samples to assess the effect of stratospheric conditions on complex microbial communities. *B. subtilis* (DSM 402; DSMZ) was grown in Luria–Bertani (LB) medium at 30°C, after which the sporulation was initiated as previously described (25). In brief, cells were plated onto nutrient agar plates containing 10 g L^−1^ MgSO_4_ and incubated at 30°C for 7 days. Endospores were harvested from the plates using an inoculation loop and washed multiple times with distilled water. The obtained endospore suspension was then pasteurized at 80°C for 20 minutes to ensure inactivation of any remaining *B. subtilis* cells. The endospore suspension was adjusted to a concentration of 10^5^ endospores mL^−1^ and microscopically checked before further use. The endospore suspension was spread onto polycarbonate plates (plate surface area = 7.5 cm^2^) in sufficiently low amounts (10^4^ endospores per plate) to allow for the formation of a single endospore layer, as calculated based on endospore density, endospore surface area, and plate surface area. The endospores were left to dry and stored at 0°C until use 1 week later. The polycarbonate plates coated with *B. subtilis* endospores were mounted at different angles to the sun (0°, 15°, 30°, and 45°) inside five replicate sample holding tubes (Fig. 1B).

The environmental samples consisted of two surface peat soil samples (0–10 cm depth; soils 1 and 2) and a lake sediment sample. The environmental samples were air-dried to a remaining moisture content of 40% prior to their use in the sample gondola (to avoid ice crystal formation in the sample holders during the flight) and stored at room temperature in the dark for 2 weeks prior to the flight. The dried soil or sediment samples was mixed well and split into control and treatment subsamples. Treatment subsamples were placed in three sterile wire cages that were stacked in one sample holding tube of the gondola (Fig. 1C). Due to limited capacity on the gondola, environmental samples were mounted in the same PMMA tube, and there were no replicates. While not completely sealed from each other, the metal cages were wedged tightly against the PMMA tube to minimize any cross-contamination between the samples. No soil material was observed in the tube space between the cages upon gondola retrieval. Earthbound control samples were stored at room temperature in the dark in 50-mL plastic tubes.

### *Bacillus subtilis* survival rates

Upon return to the laboratory after the stratospheric flight, sample holding tubes were opened, and the polycarbonate plates were aseptically removed and suspended in sterile deionized water. Endospores were detached from the plates by vigorous vortexing and the use of a sterile hydrophobic brush. The obtained spore solution was centrifuged at >37,000 rcf for 30 minutes, and the pellet was resuspended in 5 mL water. Endospore suspensions were plated onto nutrient agar in 1:1, 1:10, and 1:100 dilutions and incubated at 30°C for 48 h, after which CFUs were counted. In order to ensure that all spores had detached from the polycarbonate plates, these were incubated in the liquid nutrient broth medium at 30°C for 48 h, after which no growth was observed in the medium.

### Most probable number counts

After the flight, the number of viable aerobic and anerobic heterotrophs as well as the number of viable aerobic and anerobic metal-tolerant heterotrophs were assessed in both stratosphere-exposed and earthbound control environmental samples using an MPN approach. Serial dilutions of treatment and control samples were prepared ranging from 10^−2^ to 10^−9^. For assessment of heterotrophs, 270 µL nutrient broth medium (Sigma Aldrich) was inoculated with 30 µL in 96-well plates in six replicates per dilution step, sample, and target group (aerobic/anerobic; metal-tolerant). For assessment of metal-tolerant heterotrophs, the nutrient broth medium was supplemented with 10 mM arsenate and 10 mM arsenite. MPN plates for anerobic heterotrophs were incubated in airtight containers under an anoxic atmosphere generated by the use of AnaeroGen pouches (Thermo Fisher Scientific), while plates for aerobic heterotrophs were incubated under ambient air. All plates were incubated at room temperature in the dark. Growth of microorganisms in the plates was monitored by measuring the OD at 600 nm, and wells were scored positive if an increase in the OD was observed. MPNs were calculated based on the growth-positive wells on day 4 (general and metal-tolerant aerobic heterotrophs), day 9 (general anerobic heterotrophs), and day 15 (metal-tolerant anerobic heterotrophs). Longer incubation did not result in additional growth-positive wells.

### Community-level physiological profiling

Community-level physiological profiles of aerobic and anerobic heterotrophs were assessed with treatment (i.e., samples after stratospheric exposure) and control (i.e., samples that had remained earthbound) environmental samples in Ecoplates, which contained 31 different carbon substrates, which included carbohydrates, carboxylic acids, amines, or amino acids (Biolog, Hayward, CA, USA). Wells were inoculated with 150 µL of 10^−3^ dilution in triplicate. Aerobic heterotrophs were incubated with ambient air, while for anerobic heterotrophs, an anoxic atmosphere was generated in gas-tight containers using AnaeroGen pouches (Thermo Fisher Scientific). Color development as an indication for substrate use was determined by measurement of the OD at 590 nm in regular intervals, and color formation rates were calculated for each well in the Ecoplates.

### Microbial community composition

Microbial community composition in control and treatment environmental samples was assessed through amplicon sequencing of 16S rRNA and 16S rRNA genes. Nucleic acids were extracted from the samples using a modified phenol extraction protocol (26). Extracts were split into aliquots and treated with DNase and RNase to obtain pure RNA and DNA, respectively. RNA was reverse-transcribed to cDNA using the qScript cDNA synthesis kit (Quantabio, Beverly, MA, USA). PCR amplification of DNA and cDNA using the Earth Microbiome primer set 515F-Y/806 R (27, 28) and subsequent 250-bp paired-end sequencing on the Illumina NovaSeq platform were performed by Novogene. Demultiplexed sequence reads were imported into Qiime2 (29) for analysis. Quality filtering was conducted using the dada2 plugin (30) for paired-end sequences (“denoise_paired”), and the default parameters were modified as follows: at the 5’ end of the forward and reverse sequences, primers were removed (“--p-trim-left-f” 25 and “--p-trim-left-r” 26, respectively). Obtained ASVs were taxonomically classified using the qiime2 feature-classifier with the SILVA database version 138 as the reference. Principal coordinate analysis (PCoA) was conducted with weighted unifrac distances calculated from ASV tables rarefied to a uniform sampling depth of 15,000 sequences in Qiime2 (31).

## Data Availability

Raw amplicon sequence reads have been deposited in the European Nucleotide Archive (ENA) under accession number PRJEB70414.

## SUPPLEMENTAL MATERIAL

Supplemental figures spectrum.03982-23-s0001.pdf Fig. S1-S3.

ASM does not own the copyrights to Supplemental Material that may be linked to, or accessed through, an article. The authors have granted ASM a non-exclusive, world-wide license to publish the Supplemental Material files. Please contact the corresponding author directly for reuse.
