# Supplementary material for: Flying microbes—survival in the extreme conditions of the stratosphere during a stratospheric balloon flight experiment
Source: Microbiol Spectr. 2024 Jun 13;12(8):e03982-23. doi: 10.1128/spectrum.03982-23 (PMC11302731; doi:10.1128/spectrum.03982-23)
Supplement: Supplemental figures — Fig. S1-S3. [file spectrum.03982-23-s0001.pdf]

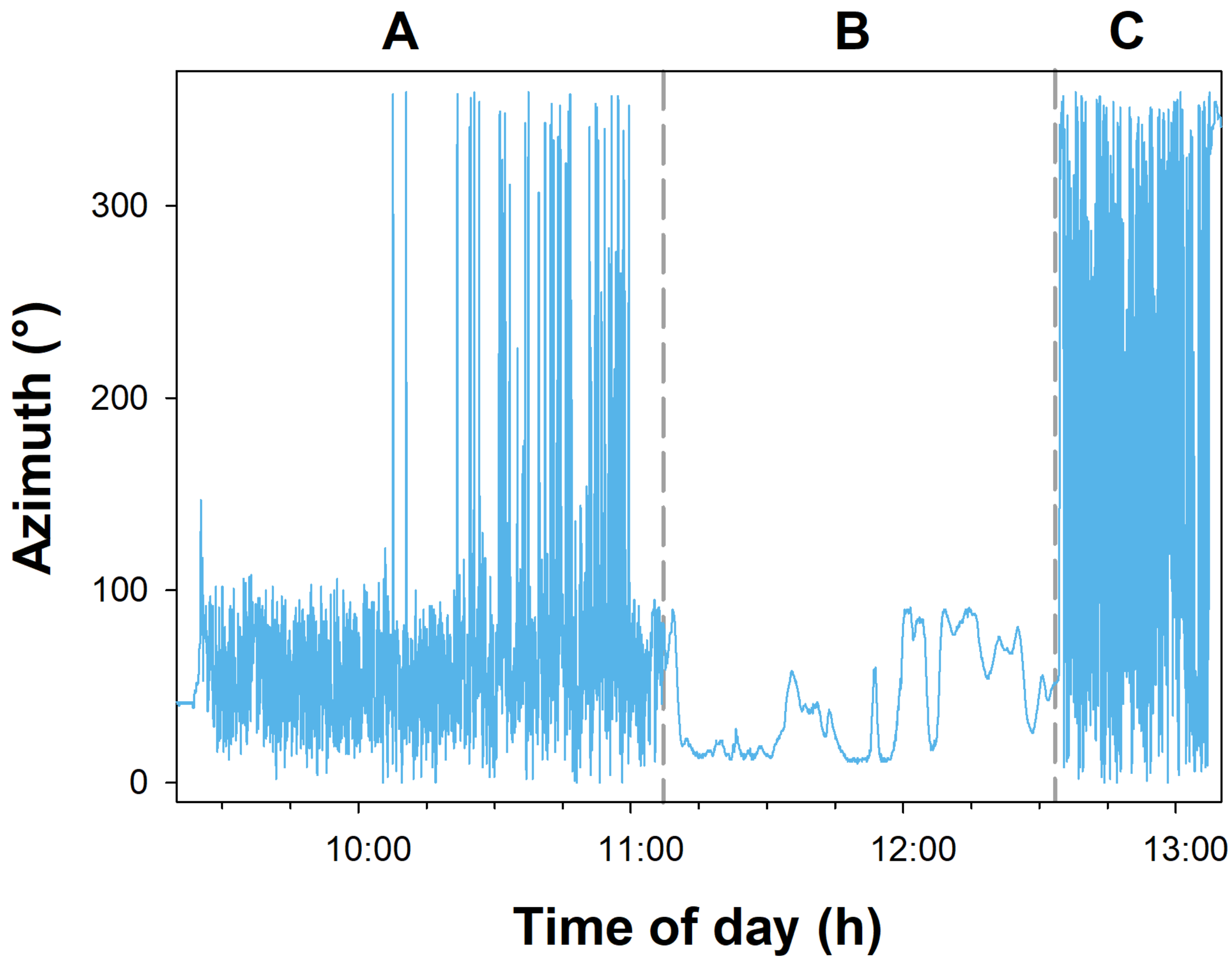

Figure S1: Angular orientation of the gondola during the flight. The different phases of the flight are indicated: A = ascent, B = floating, C = descent.

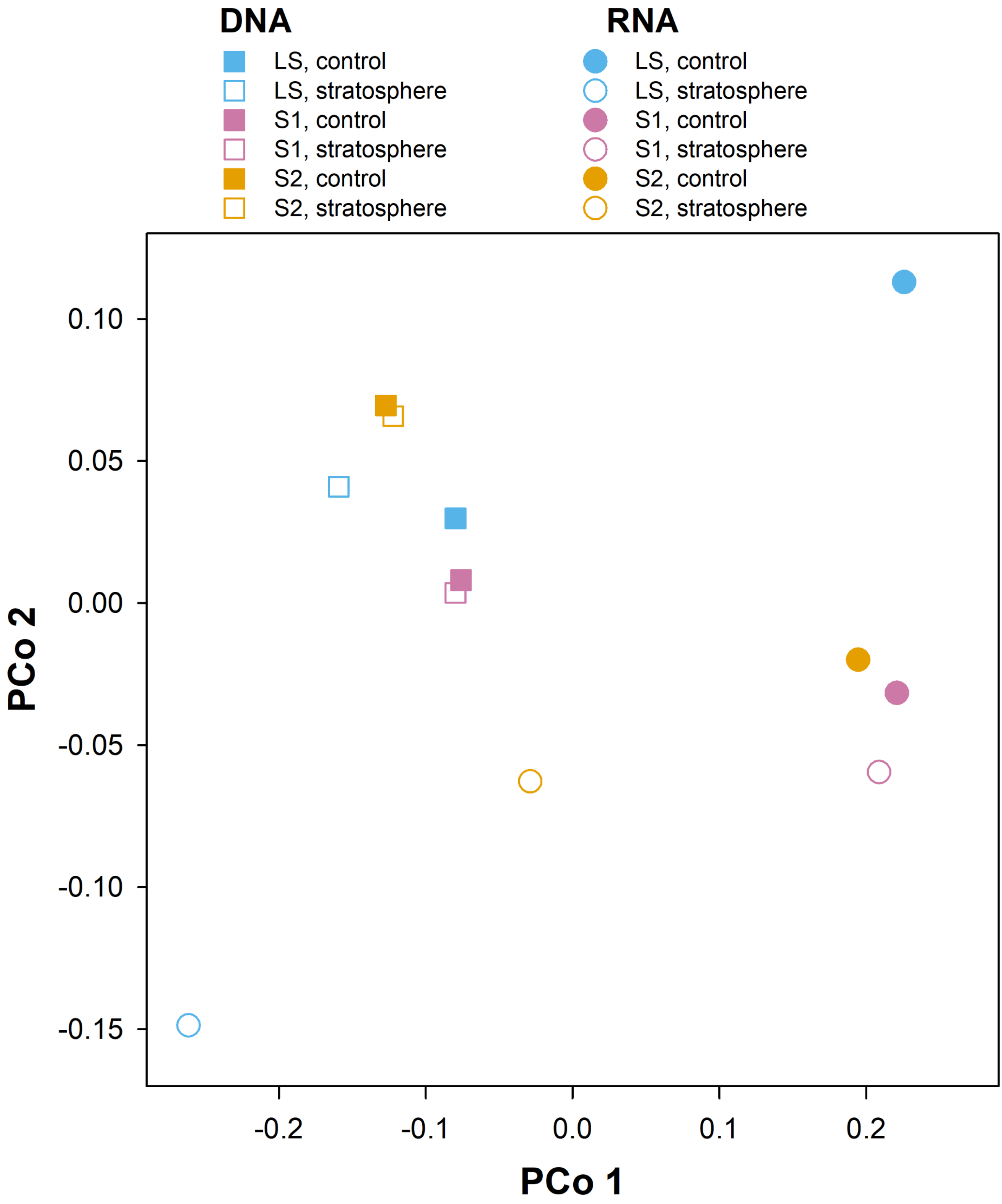

Figure S2: Impact of stratospheric flight on microbial community composition in one lake sediment (LS) and two soil (S) samples. Principal coordinate analysis based on weighted unifrac distances of ASV tables rarefied to a depth of 15,000 sequences is shown.

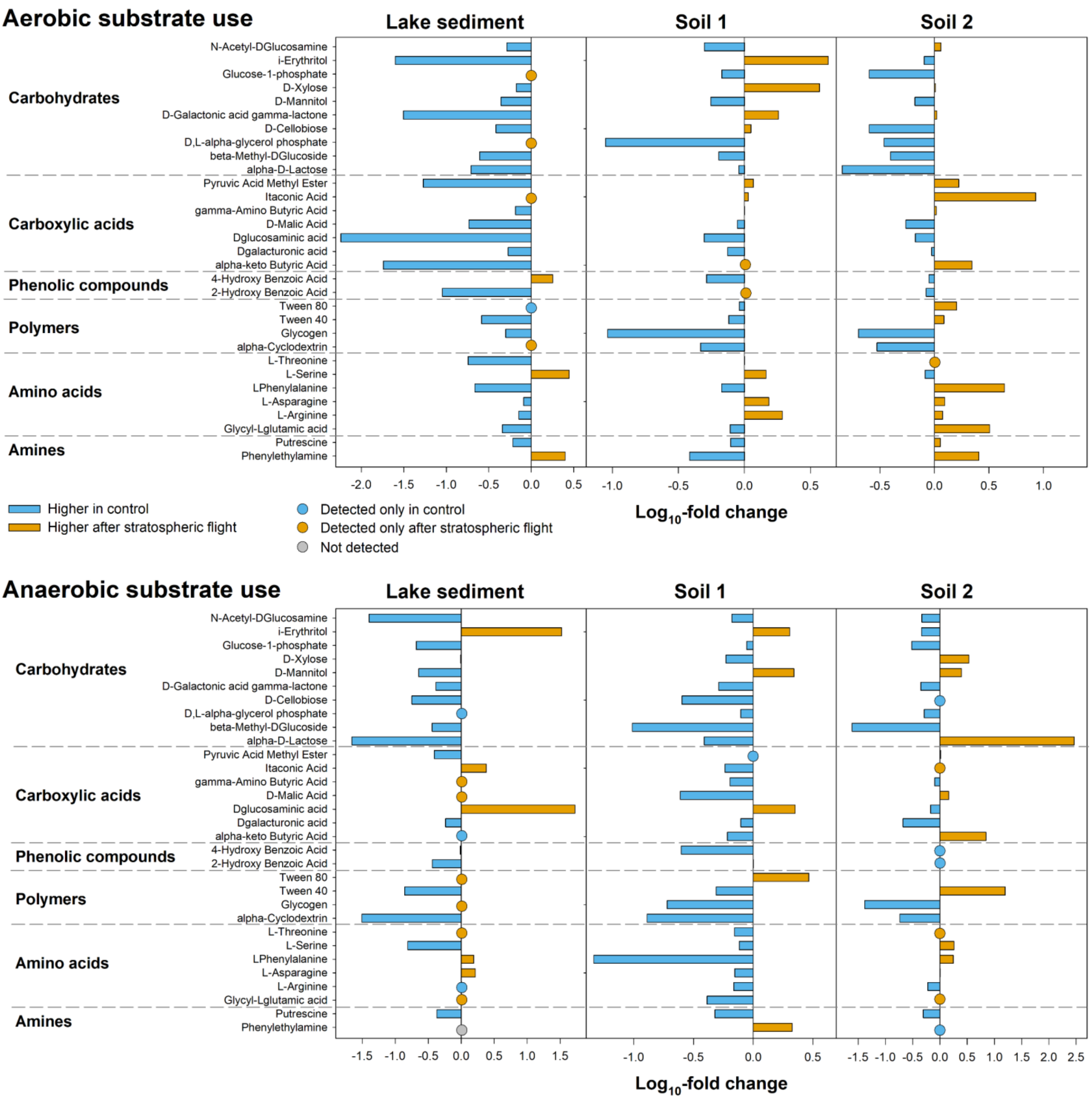

Figure S3: Impact of stratospheric flight on aerobic and anaerobic substrate use in Ecoplates. Log_{10}-fold change between the control and the stratosphere-exposed samples was calculated based on the color formation rates.
